# Laser-Treated Steel Surfaces Gliding on Snow at Different Temperatures

**DOI:** 10.3390/ma16083100

**Published:** 2023-04-14

**Authors:** Ettore Maggiore, Carmelo Corsaro, Enza Fazio, Inam Mirza, Francesco Ripamonti, Matteo Tommasini, Paolo M. Ossi

**Affiliations:** 1 Dipartimento di Chimica, Materiali e Ingegneria Chimica “G. Natta”, Politecnico di Milano, 20133 Milano, Italy; ettore.maggiore@polimi.it (E.M.); matteo.tommasini@polimi.it (M.T.); 2 Dipartimento di Scienze Matematiche e Informatiche, Scienze Fisiche e Scienze della Terra, Università degli Studi di Messina, 98166 Messina, Italy; carmelo.corsaro@unime.it (C.C.); enfazio@unime.it (E.F.); 3 HiLASE Center, Institute of Physics of the Czech Academy of Sciences (CAS), 252 41 Dolní Břežany, Czech Republic; mirza@fzu.cz; 4 Dipartimento di Meccanica, Politecnico di Milano, 20156 Milano, Italy; 5 Dipartimento di Energia, Politecnico di Milano, 20133 Milano, Italy

**Keywords:** AISI 301H, laser engraving, LIPSS, snow, friction

## Abstract

With the goal of substituting a hard metallic material for the soft Ultra High Molecular Weight Polyethylene (UHMWPE) presently used to make the bases of skis for alpine skiing, we used two non-thermodynamic equilibrium surface treatments with ultra-short (7–8 ps) laser pulses to modify the surface of square plates (50 × 50 mm^2^) made of austenitic stainless steel AISI 301H. By irradiating with linearly polarized pulses, we obtained Laser Induced Periodic Surface Structures (LIPSS). By laser machining, we produced a laser engraving on the surface. Both treatments produce a surface pattern parallel to one side of the sample. For both treatments, we measured with a dedicated snow tribometer the friction coefficient µ on compacted snow at different temperatures (−10 °C; −5 °C; −3 °C) for a gliding speed range between 1 and 6.1 ms^−1^. We compared the obtained µ values with those of untreated AISI 301H plates and of stone grinded, waxed UHMWPE plates. At the highest temperature (−3 °C), near the snow melting point, untreated AISI 301H shows the largest µ value (0.09), much higher than that of UHMWPE (0.04). Laser treatments on AISI 301H gave lower µ values approaching UHMWPE. We studied how the surface pattern disposition, with respect to the gliding direction of the sample on snow, affects the µ trend. For LIPSS with pattern, orientation perpendicular to the gliding direction on snow µ (0.05) is comparable with that of UHMWPE. We performed field tests on snow at high temperature (from −0.5 to 0 °C) using full-size skis equipped with bases made of the same materials used for the laboratory tests. We observed a moderate difference in performance between the untreated and the LIPSS treated bases; both performed worse than UHMWPE. Waxing improved the performance of all bases, especially LIPSS treated.

## 1. Introduction

The reference material for the bases of alpine skis is Ultra High Molecular Weight Polyethylene (UHMWPE; MW = 13 × 10^6^ Da; hardness 40 HB). UHMWPE is either sintered for high-level skis or extruded for lower-level equipment. In both cases, carbon-based or silicon-based particles are embedded in UHMWPE to improve the thermal and electrical conductivity of the material, obtaining a base that quickly glides on natural snow. The preparation of a ski base, both mechanically, by stone grinding, and chemically, with the use of waxes specifically designed for snows of different characteristics, increases the gliding performance of the base. The carefully sharpened edges in carbon steel (typically C60), with a smooth surface, allow the skier to easily define a precise trajectory and to maintain it along the slope.

The increasingly widespread preparation of ski slopes with *artificial* snow, much harder and more abrasive than natural snow, causes increased wear and overheating of both UHMWPE and C60 during ski gliding, especially at high speed, and produces an often severe, even irreversible damage to the base [1]. The required maintenance procedures to restore the optimal condition of a ski base are costly and time-consuming and progressively remove layers of the two materials, significantly limiting the average life of a ski. For rented equipment, the average life of a ski pair does not exceed four years. Looking at the Italian side of the Alps, a recent market survey [2] indicates that 290,000 ski pairs are available for rent from medium to large-size rentals. Each year, approximately 72,000 new pairs replace an equal number of disposed pairs. Out of these, about 35,000 are sold to less demanding markets, the remaining 37,000 being definitively disposed to landfill at a cost that is currently around 7 euros per ski pair. Such a final disposal is a process hardly compatible with environmental sustainability. Indeed, the use of strongly adhesive resins to hold together the different ski layers, made of heterogeneous materials (wood, polymers, metals), makes the selective recovery of the ski constituents difficult and at present not economically sustainable.

Metallic ski-bases could address the above concerns for the environment since they allow considerable extension of the ski durability, overcoming the intrinsic low hardness and wear resistance of UHMWPE. Any metallic alternative to UHMWPE for ski bases should significantly increase the ski average service life without loss of ski performance, as compared to that of current equipment.

During sliding on snow, the thin liquid water film obtained by frictional melting contributes to lubrication of the slider, so reducing the friction coefficient μ [3]. On wet snow, μ increases because of the capillary attraction between snow grains and the slider surface, mediated by the liquid water layer of an increased thickness [4].

The purpose of any surface treatment of the ski base is to reduce the value of the snow-base friction coefficient µ by effectively reducing the capillary force, removing the excess meltwater from the thin layer that is continuously produced by frictional heating at the snow-base contact surface [5]. For UHMWPE, stone grinding machines produce macroscopic patterns with different profiles, groove depths and spacing between them, in order to obtain the fastest gliding as a function of the snow temperature and degree of metamorphism [6]. Hot embossing of UHMWPE using stainless steel meshes of different mesh sizes was attempted to reduce the surface wetting and increase the surface hydrophobicity [7]. In the case of metal surfaces, the effect of various laser treatments to reduce the interaction with the liquid water layer was investigated [8,9]. Indeed, using laser-based techniques, the material manufacturing process is well controlled, in particular in metal additive manufacturing [10]. On these grounds, we developed conceptually new metallic bases for alpine skis, studying different materials and surface treatments [11,12].

We selected AISI 301H as a good candidate to replace UHMWPE and C60 because of its combination of elevated mechanical properties (hardness, 587.8 HB; tensile strength, 1802 MPa; yield strength, 1613 MPa), oxidation and corrosion resistance in wet environment, good workability and low cost. We treated AISI 301H square plates with ultra-short laser pulses (247 fs; 7 ps), obtaining Laser Induced Periodic Surface Structures (LIPSS) [13], and we studied the sliding behavior of such surfaces on highly compacted snow at the initial temperature of −10 °C, over a speed range up to 7.2 m s^−1^.

LIPSS is a conceptually simple, easy to control surface patterning that consists of a nearly periodic array of parallel lines produced on almost any material once it is irradiated close to the macroscopic ablation threshold. Laser engraving produces a pattern on the material surface made of a sequence of ablation craters, in part superimposed onto each other. The energy density deposited by each laser pulse must be (slightly) above the ablation threshold.

We measured the friction coefficient of steel plates on snow using a custom-made snow tribometer. For a set of tests performed at the highest initial speeds, up to 9.4 m s^−1^, the snow starting temperature was −15 °C. Overall, we observed a significant decrease of µ associated with LIPSS machining, although the reference UHMWPE waxed state-of-the-art plates perform better [13].

In this work, we move from the study carried out in [13] to explore the sliding behavior on compacted snow of AISI 301H surfaces with three different morphologies: untreated (bare) surface, laser-engraved surface, and LIPSS-treated surface. We consider the parallel and perpendicular orientation of the two laser-produced surface patterns with respect to the gliding direction of the sample on snow, and we perform the sliding tests at three initial snow temperatures (−10 °C, −5 °C and −3 °C), progressively closer to the snow melting temperature. Our choice is dictated by the observation that, in late Autumn, Winter, and early Spring, a definite trend towards increasing average temperatures was observed in the European Alps in the last 20 years [14]. Therefore, over extended periods of time, the snowpack experiences medium-high average temperatures of between −5 °C and 0 °C for several hours during the day. On approaching the melting temperature, the thickness of the quasi-liquid layer at the surface of the snow grains increases [15], their shape turns to rounded and the average size increases, with a relevant impact on the gliding properties of snow [16]. Skiers are aware of the above effects and choose significantly different grinding profiles and waxes for UHMWPE bases depending on the snow temperature.

We explore the range of relatively low sliding speeds between 1 and 6.1 m s^−1^: indeed, in this speed range, field tests performed with full scale skis equipped with bare AISI 301H bases on snow at the highest snow temperature (−3 °C) show an average µ value unacceptably higher than that of stone grinded and waxed state-of-the-art UHMWPE. Therefore, a modification of the surface morphology of bare AISI 301H appears desirable in order to improve the sliding performance of the material when sliding on snow at the often met “high” temperatures.

## 2. Experimental

Our samples were 50 × 50 mm^2^ square plates, suitable for insertion in the sample holder of the snow tribometer. The size of the samples is determined by the geometry of the snow tribometer. Along the snow track diameter, the track size is 60 mm. Parallel to the track length (tangential direction) a longer plate would lead to spurious contributions to the value of the friction coefficient, due to the track curvature. AISI 301H plates were mechanically cut from cold rolled sheets 0.5 mm thick (Lamina S.p.A., Milano, Italy). The reference stone grinded and waxed UHMWPE plate (1 mm thick) was extracted from the central section of a competition (giant slalom) ski base. The laser treatments described below were performed in air at atmospheric pressure. Before each laser treatment, we cleaned the steel surface with acetone. For the laser engraving treatment, the effectively treated plate area measured 49 × 49 mm^2^. We used a micromachining system equipped with a pulsed laser source (Coherent Rapid10), using the following experimental parameters: wavelength 532 nm, pulse duration 8 ps, laser power ~4W, repetition rate 160 kHz. The laser beam was focused on the surface of the material with a spot diameter of about 20 µm through a galvanometric scanner head equipped with a telecentric lens with a focal length of 163 mm. We obtained, on the surface of AISI 301H, parallel grooves with nominal spacing of 110 µm, from software settings. We carried out the machining with 50 repetitions for each line at a scanning speed of 10 mm s^−1^. The time to complete the sample processing was about 30 h.

We performed profilometry measurements with a stylus scanning method by using a KLA-Tencor Alpha-step 500 Surface Profiler equipped with a 5 µm tip radius with a 60° included angle (0.10 µm lateral resolution; 2.5 nm vertical resolution). The scan speed was 10 µm s^−1^ and the sampling rate was 100 Hz.

LIPSS on an AISI 301H plate were obtained with a commercial Yb:KGW laser system (PHAROS, Light Conversion) operating at a central wavelength of 1030 nm. The diameter of the irradiation spot at 1/e^2^ of peak intensity on the sample surface (as measured using the method described in [17]) was 375 µm, obtained by placing the sample ~7 mm above the focal plane of the Galvo lens. The adopted laser parameters were: 0.94 W average power, 3 kHz laser repetition rate, 313 µJ energy per pulse, 0.57 J cm^−2^ energy density of the laser, 7 ps pulse duration. A combination of λ/2 wave plate and Glan-Taylor polarizer was used to reduce the maximum available pulse energy to the required value. The overlap between the 1/e^2^ spot diameter along the X-direction was adjusted to 75% by setting the scanning speed at 0.28 ms^−1^. To obtain the same percentage overlap along the Y-direction, the distance between the scanning lines was set at 94 µm. The time to complete the processing of the plate area (50 × 50 mm^2^) was 95 s. 

We used a Zeiss Supra 40 field emission Scanning Electron Microscope (SEM), operating at the primary accelerating voltage of 5 kV to characterize the surface structure of all laser-treated samples. 

**Figure 1:**
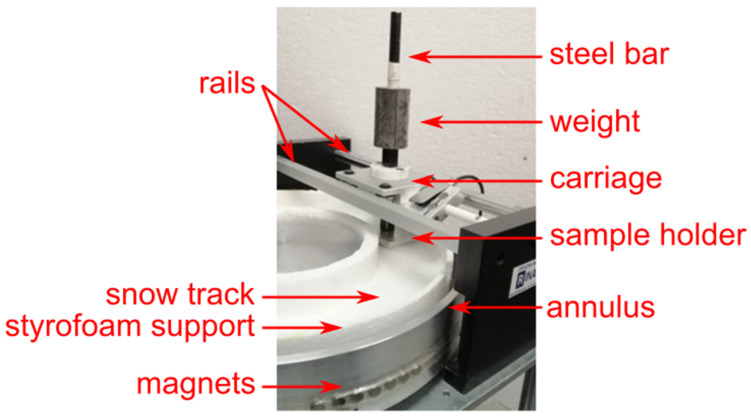
Snow tribometer with the Styrofoam container filled with snow inserted inside the aluminum annulus. The sample is held in place by the carriage and leans above the snow surface.

We measured the µ value on snow of the AISI 301H samples with different surface morphologies using a custom-built snow tribometer (Figure 1), which reproduces the sliding on snow of a ski base. The tribometer consists of an annular aluminum housing driven by a 250 W brushless electric motor connected to a controller that regulates the angular speed of the motor. The snow track is prepared separately in a polystyrene container, then inserted into the aluminum housing. The sample for the measurement of the friction coefficient is stuck to the sample holder with double-sided tape and is kept in contact with the snow surface by a steel bar and a cylindrical mass. The total mass acting on the sample (0.692 kg) was calibrated to reproduce the pressure exerted on the two skis by a skier of total mass 80 kg (including person, clothing, and equipment).

We determine the friction coefficient as a function of the sliding speed by evaluating the different slowdown times (with the motor off) that are recorded when the tribometer slows down freely with the sample gliding on the snow track. Further details on snow track preparation and analytical data treatment to extract the µ values can be found in [13].

We monitored the temperature of the snow track by permanently inserting a temperature sensor inside the track itself. For the AISI 301H samples, we carried out the tests at three initial snow temperatures: −10 °C; −5 °C; −3 °C. The slowdown tests were carried out at the ambient temperature of +13 °C. A slowdown test lasts between 48 s and 60 s, depending on the initial gliding speed. We measured the snow temperature before and after each test and we observed a maximum snow temperature increase not exceeding +2 °C, corresponding to a maximum track temperature of −1 °C when the test was performed with snow at initial temperature −3 °C. This temperature increase is the same for AISI 301H and for UHMWPE: it basically depends on the ambient temperature and on the duration of the test, namely on the initial gliding speed and on its dissipation as reflected by the μ value.

Based on the measured number of tribometer laps, we estimated that the distance traveled by the sample in a slowdown test is about 300 m. Given this short distance, the morphology of the laser-treated surfaces is not significantly affected during a test [13].

**Figure 2:**
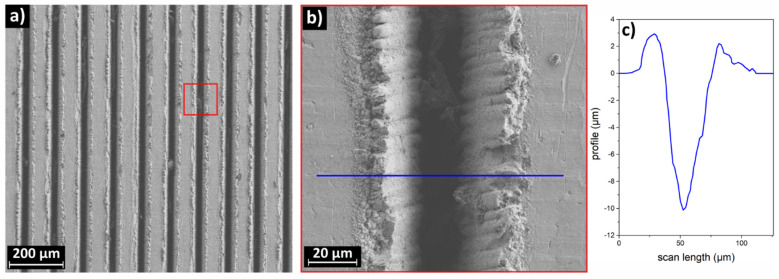
SEM micrograph of the laser engraved AISI 301H sample. (**b**) Magnification of the area marked with the red line in (**a**). (**c**) Profilometry of a representative crater.

**Figure 3:**
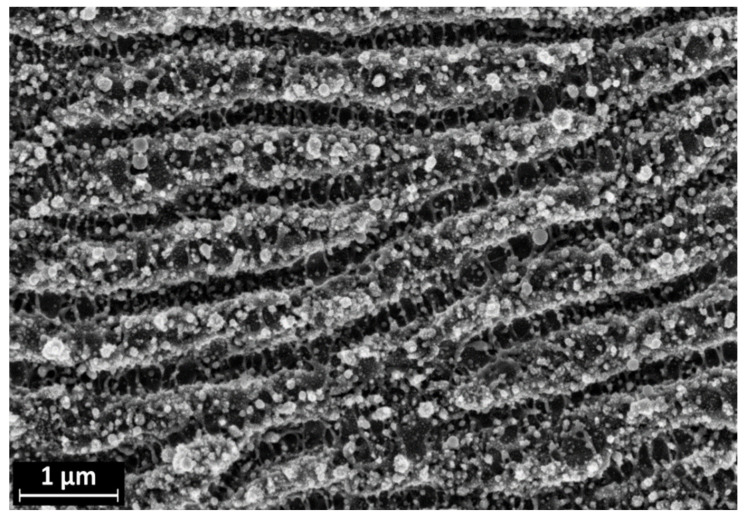
SEM image of the AISI 301H sample after LIPSS processing.

While the grooves (in the case of the laser engraved sample) are nicely aligned with each other (see Figure 2, below), ripple parallelism (in the case of LIPSS) is less precise (see Figure 3, below). For both processes, the alignment of the structures produced by the laser is parallel (roughly, for LIPSS) to one sample side. We mounted the sample on the track, either with the surface pattern lying parallel to the local direction of the running snow (parallel gliding, 0°) or perpendicular to it (perpendicular gliding, 90°).

For each combination of material, snow temperature and surface pattern orientation, we carried out two friction coefficient measurements, for which we display the resulting average µ values. For the UHMWPE, reference sample µ was measured (parallel gliding) on snow at −3 °C. At this snow temperature, bare AISI 301H has the highest µ value, consistently larger than that of UHMWPE.

## 3. Results and Discussion

AISI301H is a conventional metallic material. We chose it because it offers a convenient combination of easy workability and hardness, to withstand wear when gliding on aggressive or icy snow. It also resists oxidation when gliding on wet snow and, after the skis have been used, the metallic surface covered by water droplets is exposed to ambient air. 

Our choice of the laser engraving and LIPSS treatments for AISI 301H depends on the highly non-thermodynamic equilibrium nature of such surface modification processes. We expect that both treatments result in modification of the tribological properties of the irradiated surfaces with respect to the bulk AISI 301H beneath.

Just above the ablation threshold, a few nominally identical pulses that illuminate the same area are enough to produce a crater. The average crater width is controlled by the laser spot size, the depth by the deposited energy density per pulse and by the number of laser pulses. Introducing the movimentation of the beam on the target surface, engraves made of sequences of craters adjacent and partly superimposed to each other are produced. Several lateral displacements of the beam with respect to a first engrave allow the drawing of further engraves, nicely parallel to the first one. In Figure 2, we display a representative SEM micrograph of the laser engraving on the AISI 301H surface.

Figure 2a shows the engravings generated by the laser processing. We measured an average spacing of 105 µm between the centers of two adjacent engraves. The morphology of the material redeposited laterally on both sides of the engrave indicates that it is due to the fast melting and redeposition of the material. The bottom of the engrave shows no macroscopic asperities along consistent lengths (in Figure 2b about 130 µm from the top to the bottom of the picture, along the crater). Figure 2c shows the typical lateral profile of an engrave, as obtained by profilometry measurements. The side profile of the walls is slightly flared, the width of the engraving is comparable with the diameter of the laser spot (20 µm) and the maximum depth of the engrave is about 10 µm. Since, along the engravings, matter is ablated, a related local surface compositional change, possibly associated to oxidation, could occur.

Pulsed Laser Ablation (PLA) is based on non-thermodynamic equilibrium mechanisms. The interaction of a ns and a laser pulse of a few ps with the surface of a solid target leads to several subsequent steps, from ultrafast (timescale in the fs range) target excitation to melting, evaporation, formation of a dense vapor cloud above the laser spot, absorption of laser energy, and conversion to an expanding plasma with estimated electron temperature T_e_ in the range 1–10 eV. On the target side, in the case of a metallic material, the initial excitation involves the electronic channel. Only on the ps timescale can electron-phonon coupling activate lattice conduction and energy dissipation towards the initially unperturbed crystalline lattice. The onset of a shockwave towards the target bulk is often observed because of the sudden detachment from the target surface of the highly energetic plasma that is normally strongly accelerated to the surface itself. A further consequence is the possible formation of a laterally lifted profile of the ablation crater following bouncing of the melted material.

LIPSS are self-ordered, nearly regular structures, with nano/micro-metric size that can be generated on the surface of metals [18], polymers [19], and ceramics [20] upon irradiation with polarized laser radiation, at energy density values near the ablation threshold of the material [21]. By LIPSS, it is possible to modify the tribological [22] and wettability [23] properties of a surface. 

In the case of our concern with linear polarization, after laser treatment of AISI 301H we observe a macroscopic surface pattern made of narrow, nearly regular lines with a spacing close to the laser wavelength (Low Frequency LIPSS, LSFL). This kind of “classical” LIPSS has been observed since the 1980s, using pulses as long as nanoseconds. In strongly absorbing materials like metals, the orientation of LSFL is usually normal to the direction of the laser polarization. In terms of process control, the average length of the generated ripples is proportional to the pulse eccentricity, given by the semi-axes of the polarization ellipse.

The origin of LSFL is the interaction of the incident beam with an electromagnetic wave scattered at the non-ideally flat target surface, as corrugated by the first laser pulse. The excitation of collective electron modes, typically Surface Plasmon Polaritons (SPP), is likely to occur. With linearly polarized ultra-short laser pulses, LSFL remains the dominating morphological feature produced on the surface of metals. With pulses in the few ps to few tens of fs range, “non-classical” LIPSS, or nanoripples, are also observed. These High Spatial Frequency LIPSS (HSFL) consist of lines featuring around 10^2^ nm, and thus much narrower than LSFL, well below the diffraction limit. HSFL are usually oriented parallel to the polarization direction.

The morphology of LIPSS treated AISI 301H surfaces is reported in Figure 3. It consists of a pattern of roughly parallel channels of mean width ~200 nm (LSFL). HSFL are visible across the channels, perpendicular to the LSFL direction. A distribution of nearly spherical nanoparticles with an average diameter of 70–80 nm is also evident. The periodicity of the LSFL is around 700 nm, comparable with the wavelength of the laser (1030 nm). We used the Gwyddion software, version 2.62 to calculate the average distance between the ripples on the LIPSS surface. After LIPSS treatment some oxidative effects were reported (e.g., in Ti6Al4V after LIPSS formation upon irradiation of the surface with femtosecond laser pulses [24]). Our choice of AISI 301H and of a fluence value below the ablation threshold makes oxidative processes associated with laser irradiation highly unlikely. The duration of ultra-short pulses and the associated target excitation is much faster than all relaxation mechanisms: during a single pulse, a target atom at thermal speed can travel less than a lattice spacing, thereby the intrinsic target dynamics are decoupled from the excitation.

In the simple case of a metal, or a narrow gap semiconductor, absorption of the laser pulse brings about strong excitation of the electron gas that results in the softening of the crystalline bonding, with the associated formation of an intermediate state of matter aggregation, intermediate between liquid and solid. Atoms are free to explore “large” distances, while still loosely bound to their equilibrium sites. Such an excited state rapidly relaxes, giving rise to a self-organization of the irradiated material to release the surface instability originated by surface erosion and atomic diffusion, the very same mechanisms that drive surface topography changes upon energetic ion bombardment of a solid target. HSFL are the final output of the relaxation process. Although there is current debate about the different mechanisms concerning the origin of HSFL, ultra-fast non-equilibrium energy deposition at the target surface and the subsequent relaxation appear to be relevant. The lower limit, around 50 nm, observed for the HSFL spatial periodicity is likely to be a consequence of the thermal diffusivity of the target that cancels small spatial modulations on the surface when energy is transferred at “long” times from the electronic channel to the phonon channel, a mechanism driven by the thermal conductivity of the target. 

**Figure 4:**
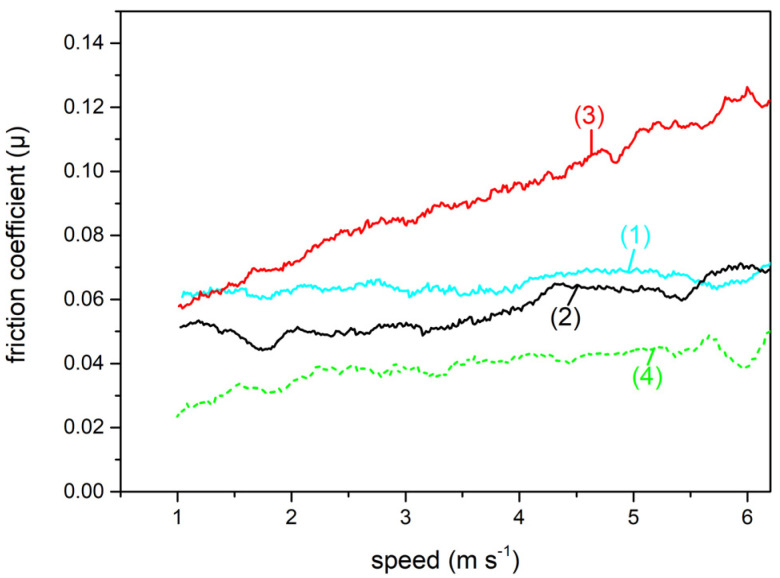
Friction coefficient of bare AISI 301H as a function of the gliding speed on snow at different temperatures: −10 °C (cyan curve, 1); −5 °C (black curve, 2); −3 °C (red curve, 3). For comparison, the µ trend for parallel gliding at −3 °C of stone grinded and waxed UHMWPE (dashed green curve, 4) is shown.

**Figure 5:**
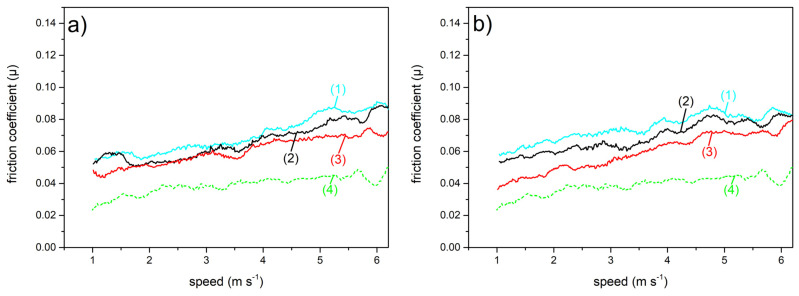
Friction coefficient of AISI 301H, laser engraved, as a function of the gliding speed on snow at different temperatures: −10 °C (cyan curve, 1); −5 °C (black curve, 2); −3 °C (red curve, 3). For comparison, the µ trend for parallel gliding at −3 °C of stone grinded and waxed UHMWPE (dashed green curve, 4) is shown; (**a**) parallel gliding; (**b**) perpendicular gliding.

**Figure 6:**
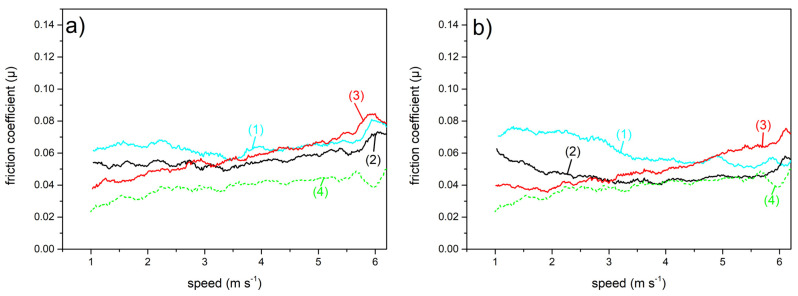
Friction coefficient of AISI 301H LIPSS as a function of the gliding speed on snow at different temperatures: −10 °C (cyan curve, 1); −5 °C (black curve, 2); −3 °C (red curve, 3). For comparison, the µ trend for parallel gliding at −3 °C of stone grinded and waxed UHMWPE (dashed green curve, 4) is shown; (**a**) parallel gliding; (**b**) perpendicular gliding.

We now report and discuss the results of the µ measurements of the differently surface treated AISI 301H samples. Before entering the details of the measurements, we notice that all the μ vs. gliding speed curves (see Figure 4, Figure 5a,b and Figure 6a,b below) show fluctuations. These are intrinsic to our experiments, and they depend on the small surface irregularities that can accidentally be present on the snow track. Figure 4 shows the friction coefficient of bare AISI 301H as a function of the gliding speed. For this surface treatment, since there are no patterns on the sample surface, the orientation of the surface with respect to the direction of the running snow is irrelevant. 

At −10 °C (cyan curve, 1), µ is almost constant at the value of about 0.06. For a quick comparison, this value is near the value measured for stone grinded and waxed state of the art UHMWPE, at this same snow temperature. At −5 °C (black curve, 2), µ values weakly increase between 0.05 and 0.06 with increase in the gliding speed. At −3 °C (red curve, 3), apart from the lower speeds where µ (0.06) has a value comparable with the values observed for the lower temperatures, we observe a significant progressive increase of µ with the gliding speed, up to 0.12. The severe deterioration of gliding ability of bare AISI 301H on high temperature snow confirms that a surface treatment is vital to obtain acceptable performance of the ski base at such (and higher) marginal snow temperatures.

Figure 5a,b refer to the µ values of laser engraved AISI 301H. Overall, we note a weak increase in the friction coefficient with speed at all temperatures and for both gliding orientations (parallel, Figure 5a; perpendicular, Figure 5b) of the surface with respect to the direction of the running snow.

**Figure 7:**
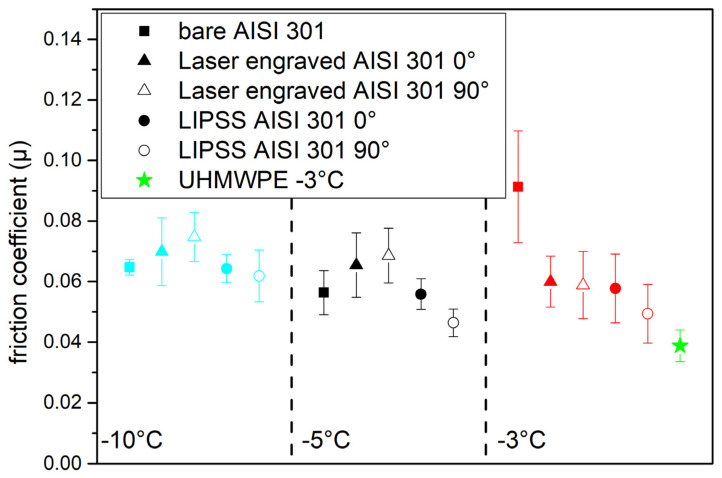
Average µ values of differently treated AISI 301H samples, at different temperatures and for parallel (full symbols) and perpendicular (open symbols) gliding on snow. For reference the green star is the average µ value for parallel gliding at −3 °C of stone grinded and waxed UHMWPE.

With increasing gliding speed, we do not observe significant differences in the µ values related to the parallel versus the perpendicular orientation of the glider. At each temperature, the slopes of the curves and the amplitude of the oscillations in the μ values are also comparable to each other for the two orientations. We observe that, on average, at each temperature, at speeds lower than 3 m s^−1^, µ values for perpendicular gliding are slightly lower than for parallel gliding. This result can be better appreciated from Figure 7. 

Figure 6 shows the friction coefficient as a function of the gliding speed on snow of the LIPSS treated AISI 301H sample, again for the two gliding orientations (parallel, Figure 6a; perpendicular, Figure 6b).

In both cases at low sliding speed values (less than 3 m s^−1^), µ depends more strongly on the snow temperature than at higher speeds. At −10 °C and −5 °C for parallel gliding, µ is almost constant, with a slight increase at the higher gliding speeds of above 5.5 ms^−1^. For perpendicular gliding, µ values after an initial slight decrease with increasing gliding speed, show a trend to increase at the higher speeds. The trend is less marked than for parallel gliding. At −3 °C for both orientations, the friction coefficient smoothly increases as the gliding speed increases, similarly to what we observed for the laser engraved surface.

Figure 7 displays the average µ values with the relative dispersions of the differently treated AISI 301H samples. The acceptably low µ values of bare AISI 301H at low snow temperature (−10 °C and −5 °C) confirm the gliding speed data recorded on field tests, where full scale ski pairs identical to each other, except the base, bare AISI 301H and stone grinded UHMWPE waxed state-of-the-art, respectively were compared to each other [25].

We observe that, at low temperatures (−10 °C), all μ values lie in the narrow range between 0.065 and 0.075, irrespective of the surface treatment. With respect to bare AISI 301H, laser engraving results in a slight worsening of μ values, while LIPSS brings to nearly constant (at −10 °C) or slightly reduced (at −5 °C) μ values. At −3 °C, the initial μ value for bare AISI 301H significantly reduces, both after laser engraving and after LIPSS. Both at −3 °C and at −5 °C, the LIPSS treated surface subjected to perpendicular gliding shows the lowest μ values (open black and red dots) that approach much the same reference value as properly waxed, stone grinded UHMWPE (green star), with nearly the same performance increase, with respect to parallel gliding. Such results are promising, since the metallic samples were not waxed (current waxes are not conceived for any metallic material). Indeed, waxing materials and procedures have been specifically developed over the years for UHMWPE. 

By contrast, μ values of laser engraved surfaces (triangles) appear not sensitive to the gliding orientation (at −3 °C), while parallel gliding performs better than perpendicular gliding at the lower temperatures, an opposite trend to that of the LIPSS treated surfaces. Overall, the LIPSS treated sample shows lower µ values than the laser engraved sample.

The modest effect of surface treatments on the gliding properties of AISI 301H at low snow temperature (−10 °C) (µ values are similar for all samples) depends on the reduced thickness of the liquid water layer [26] that transiently forms under the gliding surface, due to the combined effect of the pressure exerted by the simulated skier and of friction melting [27]. At a high snow temperature (−3 °C), the liquid water layer is considerably thicker than that formed at lower snow temperatures. The resulting capillary drag critically affects the gliding of the smooth surface of bare AISI 301H and gives rise to a progressive increase in µ value with increasing gliding speed. The surface corrugation artificially produced by laser processing breaks the continuity of the quasi-liquid layer and reduces the extent of capillary drag. At −3 °C, the slight µ decrease associated with perpendicular gliding, with respect to parallel gliding, is likely to mirror the better performance of herringbone grinding, as compared to parallel grinding observed in UHMWPE bases.

For both laser treatments, after completing the μ measurements, we did not observe any modification of the surface morphology of the samples. This was not surprising. Indeed, in the past we observed that, after a distance travelled of 10 km, LIPSS are preserved on the surface of LIPSS treated AISI 301H samples, and only local damage such as partial flattening of the ripples and the occurrence of shallow scratches occurred [13].

We complemented the above laboratory tests by field tests to estimate the gliding ability of full-size skis. Field tests were performed on snow with a high liquid water content, undergoing fast, extensive metamorphism under the combined action of elevated air temperature and humidity. In these ambient conditions, metallic bases are expected to show the worst performance, with a large increase in gliding friction, with respect to UHMWPE bases [25]. The tests were performed on 9 March 2023 in Gressoney St. Jean (Aosta Valley, Italy) on a slope with gentle, constant inclination, following the general recommendations for performing such tests. The length of the rectilinear test track was 60 m, the altitude was 1387 m a.s.l., and the difference in altitude between the start and the arrival was 3 m. The night before the test, the slope was subjected to heavy rain, followed by a weak snowfall (thickness of the deposited fresh layer, 3.5 cm). The tests were performed on a sunny day, at an initial (8.30AM) air temperature of +3 °C, with relative humidity of 88%. The initial snow temperature was −0.5 °C. The snow was wet, with rounded, sintered grains. With elapsing time, the snow temperature increased to 0 °C, and the air temperature increased to +7 °C (at 1PM), with relative humidity of 85%. The test track was initially exposed to sun irradiation along its whole length, then at later times about one fourth of the track length was shadowed. Two professional testers used three pairs of skis identical to each other, made by Blossom Skis (Prata Camportaccio, Italy) according to the present standards released by the International Ski and Snowboard Federation (FIS) on the size (length, sidecut) of skis for Giant Slalom competition. The bases of the three pairs of skis were made of three materials: standard stone grinded UHMWPE (reference), bare AISI301H, and AISI301H LIPSS treated with the same laser treatment used to prepare the laboratory plates (processing time, 20 min. for each base). The LIPSS treatment on the ski bases was performed at Kirana srl (Rovereto, Italy). The mass of the testers, including skis, bindings and poles, was measured for each pair of skis before beginning the tests. According to the standard procedure, the tester crosses the gate without doing any voluntary muscular action and stays on the skis until he crosses the photoelectric cell at the arrival, and then stops. The time needed to complete the track is automatically recorded. In Table 1, we display the times collected for the tests performed by the two testers using the skis with different bases. Looking at the data, the much larger Tester 1 (101.5 Kg) is always faster than Tester 2 (83.5 kg). Notice that Tester 1 kept an aerodynamic position on the skis during the tests, while Tester 2 kept a base position, thus exposing a larger body surface to air. The time marked with the asterisk in Table 1 refers to the very first descent on the slope, along which the fresh snow layer was compacted, thus producing the ski rails along which all successive tests were run.

A second set of tests was performed (initial time, 11.30AM) after waxing the bases with a commercial liquid sprayable wax (MAPLUS FP4) intended for snow temperatures between −3 °C and 0 °C. The wax is suited for UHMWPE, and was applied to the UHMWPE base following the standard procedure. On the metallic bases, we sprayed the wax, waiting for the evaporation of the liquid phase. After the evaporation stage, a white finely grained dust remained on the metallic ski bases as an uneven deposit.

Looking at the data in Table 1a, unwaxed UHMWPE performs better than AISI 301H, both bare and LIPSS treated. Overall, the waxed bases glide faster than the untreated bases (see the average travel times in (a) of Table 1). As evident from (b) of Table 1, which reports the travel time differences with respect to UHMWPE (taken as reference), there is no performance difference between the two unwaxed metal bases for Tester 1, while for Tester 2 the AISI 301H bare base performs better than the LIPSS treated base. From (b) of Table 1, waxing reduces the performance difference between UHMWPE and AISI 301H (both bare and LIPSS treated) in the case of Tester 1. For Tester 2, waxed metallic bases slightly outperform, with respect to UHMWPE.

## 4. Conclusions

In conclusion, we measured the friction coefficient μ on snow at different temperatures of differently laser treated AISI 301H plates. We produced a surface pattern aligned along a preferential direction: one consisting of parallel engravings, the other of ripples nearly parallel to each other (LIPSS). The comparison between the µ values on snow of the laser treated samples and of bare AISI 301H shows that both surface patterns display appreciable lower µ values at the highest snow temperature we tested (−3 °C). The effect of the pattern orientation with respect to the speed of the gliding snow (parallel or perpendicular) is small, but noticeable. Field tests on full-size skis with bases made of AISI 301H (bare and LIPSS treated) showed appreciable differences in performance with respect to UHMWPE bases. For unwaxed metal bases, the performance is worse than that of unwaxed UHMWPE. However, the performance of waxed metal bases approaches that of waxed UHMWPE and may be even slightly better. Considering that the gliding ability of metal bases worsens at low speeds and on snow at high temperature, possibly undergoing metamorphism, we consider the present results promising in view of the preparation of waxes specific for metal bases.

From the perspective of the treatment of a full-scale ski surface, the time required for laser processing is a crucial factor. The much shorter time required to carry out LIPSS with respect to laser engraving on samples with a small surface (50 × 50 mm^2^) (see Section 2) indicates that the former is likely to be preferable.

## Figures and Tables

**Table 1:** (**a**) Collection of travel times (t, seconds) for the two testers equipped with the three different skis. The total mass of Tester 1 was 101.5 kg and that of Tester 2 was 83.5 kg. (**b**) Difference (Δt, seconds) of the average travel times recorded for AISI301H ski bases with respect to UHMWPE. * see text for details.

(a)						
t (s)	Unwaxed	Unwaxed	Unwaxed	Waxed	Waxed	Waxed
	AISI301H-bare	AISI301H-LIPSS	UHMWPE	AISI301H-bare	AISI301H-LIPSS	UHMWPE
**Tester 1**	8.40	8.52	7.90	7.63	7.66	7.40
		8.25	7.50	7.55	7.53	--
**average**	8.40	8.39	7.70	7.59	7.60	7.40
**Tester 2**	8.40	9.10 *	8.00	8.00	8.10	8.10
	8.52	8.70	8.30		7.76	8.01
	8.30		8.20			
**average**	8.41	8.90	8.17	8.00	7.93	8.06
**(b)**						
**Δt (s)**	**Unwaxed**	**Unwaxed**	**Waxed**		**Waxed**	
	**AISI301H-bare**	**AISI301H-LIPSS**	**AISI301H-bare**		**AISI301H-LIPSS**	
**Tester 1**	0.70	0.69	0.19		0.26	
**Tester 2**	0.24	0.83	−0.05		−0.13	

## Data Availability

The data presented in this study are available on request from the corresponding author.
